# A Study of Remitted and Treatment-Resistant Depression Using MMPI and Including Pessimism and Optimism Scales

**DOI:** 10.1371/journal.pone.0109137

**Published:** 2014-10-03

**Authors:** Masatoshi Suzuki, Michio Takahashi, Katsumasa Muneoka, Koichi Sato, Kenji Hashimoto, Yukihiko Shirayama

**Affiliations:** 1 Department of Psychiatry, Teikyo University Chiba Medical Center, Ichihara, Japan; 2 Division of Clinical Neuroscience, Chiba University Center for Forensic Mental Health, Chiba, Japan; Institute of Psychiatry, United Kingdom

## Abstract

**Background:**

The psychological aspects of treatment-resistant and remitted depression are not well documented.

**Methods:**

We administered the Minnesota Multiphasic Personality Inventory (MMPI) to patients with treatment-resistant depression (n = 34), remitted depression (n = 25), acute depression (n = 21), and healthy controls (n = 64). Pessimism and optimism were also evaluated by MMPI.

**Results:**

ANOVA and post-hoc tests demonstrated that patients with treatment-resistant and acute depression showed similarly high scores for frequent scale (F), hypochondriasis, depression, conversion hysteria, psychopathic device, paranoia, psychasthenia and schizophrenia on the MMPI compared with normal controls. Patients with treatment-resistant depression, but not acute depression registered high on the scale for cannot say answer. Using Student's t-test, patients with remitted depression registered higher on depression and social introversion scales, compared with normal controls. For pessimism and optimism, patients with treatment-resistant depression demonstrated similar changes to acutely depressed patients. Remitted depression patients showed lower optimism than normal controls by Student's t-test, even though these patients were deemed recovered from depression using HAM-D.

**Conclusions:**

The patients with remitted depression and treatment-resistant depression showed subtle alterations on the MMPI, which may explain the hidden psychological features in these cohorts.

## Introduction

Approximately 80 to 90 percent of depressed patients respond to antidepressant treatment, while the remaining 5 to 15 percent are deemed non-responders. Treatment-resistant depression is defined as a non-response to at least two types of antidepressant medication [1], [2]. A response is defined as a reduction in depressive symptoms to less than 50 percent while remission means a full recovery.

The Minnesota Multiphasic Personality Inventory (MMPI) is a screening instrument for the differentiation of various forms of psychopathology [3]. Personality is considered to be a modifier for depression. It is not surprising that high depression scores are found to be modestly accurate in predicting depression [4]–[6], as it is well known that a high score on the MMPI depression scale is not specific to depression [7]. A previous study showed that the MMPI profile for patients with unipolar depression was characterized by an elevation on the depression scale and a secondary elevation on the schizophrenia scale, while the MMPI profile for the bipolar depression group showed comparable elevations on both depression and schizophrenia scales [8]. Another study showed small but significant differences between dysthymia and major depression, but the presence of a personality disorder increased the neurotic triad, namely the hypochondriasis, depression and hysteria scales [9]. Similarly, comorbidity of a personality disorder with major depression increased schizophrenia scores, while major depression without a personality disorder increased psychasthenia scores on the MMPI [10]. Furthermore, when scoring depressed patients on the MMPI, the high scoring scales excluding depression, were hysteria, psychopathic deviation, schizophrenia and psychasthenia [11]. Therefore, on the MMPI, combinations of scores excluding depression may be useful for detecting the psychological mechanisms involved in remitted and treatment-resistant depression.

Interestingly, a previous study demonstrated that scores on the MMPI were useful for evaluating pessimism and optimism [12]. This scale was developed on the basis of a working hypothesis. The hypothesis states that the manner in which people attempt to understand the causes of a stressful or adverse life event can significantly undermine their psychological and physiological function or adversely affect the course of a disease [13], [14]. Peterson and Seligman put forward that a pessimistic explanatory style could predict an escalation in depressive symptoms [13]. A recent study reported pessimistic personality traits increased all-cause mortality [15].

The present study attempts to explore the psychological features underlying both treatment-resistant and remitted depression (single episode) using the MMPI. We administered the MMPI to patients with treatment-resistant depression, remitted depression and acute depression, and compared them with normal healthy controls. In addition, we evaluated the pessimism or optimism scores in these same cohorts of patients using the MMPI.

## Methods

### Ethics Statement

The study was approved by the ethics committee of Teikyo University Chiba Medical Center (study number 09–30), and performed in accordance with the Declaration of Helsinki. Written informed consent was obtained from all participants after a full explanation of all procedures.

### Participants

Sixty four healthy subjects, 25 remitted depressed patients, 21 acutely depressed patients and 34 antidepressant treatment-resistant depressed patients were enrolled in this study (**Table 1**). All patients met the DSM-IV criteria for major depressive disorder (first episode) [16]. All patients were recruited from the outpatient clinics of Teikyo University Chiba Medical Center. Two senior-level psychiatrists assessed the psychopathology of patients with treatment-resistant depression. Participants were physically healthy and free from alcohol or drug abuse. Inclusion criteria for treatment-resistant depression required symptoms of moderate depression after treatment with at least two antidepressants, for eight weeks. Patients with treatment-resistant depression had typically received at least two antidepressants, whereas acutely depressed patients were medication-free or receiving their first antidepressant trials. The remitted group received one or two antidepressants trials (Table 1). The depression scores of patients with acute depression and treatment-resistant depression were 14 or more on the 17-item Hamilton Rating Scale for Depression (HAM-D), on which, the definition of remission (recovery) was defined as 7 or less in patients with remitted depression [1], [2] (Table 1). Only patients who had suffered a single depressive episode were included in the study. The controls were healthy volunteers recruited from hospital staff, their associates and friends. Those who had a current or past history of psychiatric treatment or drug dependence were not enrolled in this study. All controls were employed men or women.

**Table 1:** Demographic information for subjects.

	Healthy Control	Remitted Depression	Acute Depression	Treatment-resistant Depression	P values
	n = 64	n = 25	n = 21	n = 34	
Current age (years)	37.47±8.46 (24–53)	40.08±9.92 (22–58)	40.19±10.79 (23–58)	39.03±9.48 (22–53)	0.526
Sex (male/female)	54/10	18/7	16/5	24/10	0.370
Age at onset (years)		36.56±9.65 (22–54)	39.81±10.93 (23–55)	36.15±8.99 (17–50)	0.369
Duration of depressive state (months)		23.70±20.67 (4–68)	2.24±3.07^a^ (1–7)	37.15±21.24^a^^,^ b (9–98)	<0.001
Duration of treatment (months)		26.88±26.68 (6–54)	0.14±0.64^a^ (0–1)	30.65±21.30^a^^,^ b (4–97)	<0.001
HAM-D		4.36±1.73 (3–7)	20.62±4.27^a^ (16–30)	18.35±4.04^a^ (14–28)	<0.001
Trial numbers of antidepressants		1.28±0.45 (1–2)	0.05±0.21^a^ (0–1)	2.50±1.26^a^^,^ b (2–9)	<0.001

Data are shown as mean ± SD.

HAM-D: Hamilton Rating Scale for Depression

a P<0.001 as compared to the remitted group.

b p<0.01 as compared to the acute depression group.

Parenthesis denotes the range.

### Personality evaluated by MMPI

MMPI is used worldwide to assess patient personality types [3]. For the purpose of shortening procedural times, we used the short version of the MMPI, which consists of 383 questions and 13 domains (4 validity scales and 9 clinical scales). MMPI data provides a standardized and quantitative measure of personality traits. Validity scales involve the cannot say answer scale (?), the lie scale (L), the frequent scale (F), and correction scale (K). The clinical scales detect the presence of psychopathological features, namely, hypochondriasis (scale 1), depression (scale 2), hysteria (scale 3), psychopathic device (scale 4), paranoia (scale 6), psychasthenia (scale 7), schizophrenia (scale 8), hypomania (scale 9) and social introversion (scale 0). Scale 5 (masculinity-femininity) was not used since the participants included both males and females.

A raw score on the can not say answer scale greater than 30 was considered grounds for declaring a profile invalid. A total of six MMPIs data were discarded for this reason.

### MMPI for pessimism and optimism

Briefly, the habit of voicing pessimistic or optimistic explanations for the cause of a stressful or adverse life event can undermine their psychological functioning [13] or adversely affect the course of a disease [14]. Pessimism and optimism were evaluated using the methods of a previous study [12]. Half of the MMPI items described good or bad, both of which mirrored various life experiences. Thus, this scale is capable of assessing a patient's beliefs around the cause of good and bad events, based on the pattern of item endorsement.

Since the short version of MMPI is used to reduce clinical procedural time, we checked coefficients between the 550 original and 383 short versions of the MMPI in the same participants, using Pearson coefficient (n = 20). The coefficients of pessimism and optimism were 0.9919 and 0.9488, respectively (both P values were less than 0.0001), validating the short version of MMPI for pessimism and optimism.

### Statistical Analysis

Data from 13 domains (4 validity scales and 9 clinical scales) of the MMPI were first analyzed using multiple analysis of variance (MANOVA) to confirm the ability to determine the simultaneous, significant differences. Statistical differences among the four groups were determined by a one-way factorial analysis of variance (ANOVA), followed by multiple comparison testing (Scheffe's test). Statistical evaluation between the two groups was performed using two-tailed Student's t-test or Chi-square test. Differences were considered to be significant when p values were less than 0.05.

## Results

MANOVA for all scales (validity and clinical scales) indicated a significant effect for group (F = 7.154, P<0.0001).

### MMPI for validity

**Figure 1:**
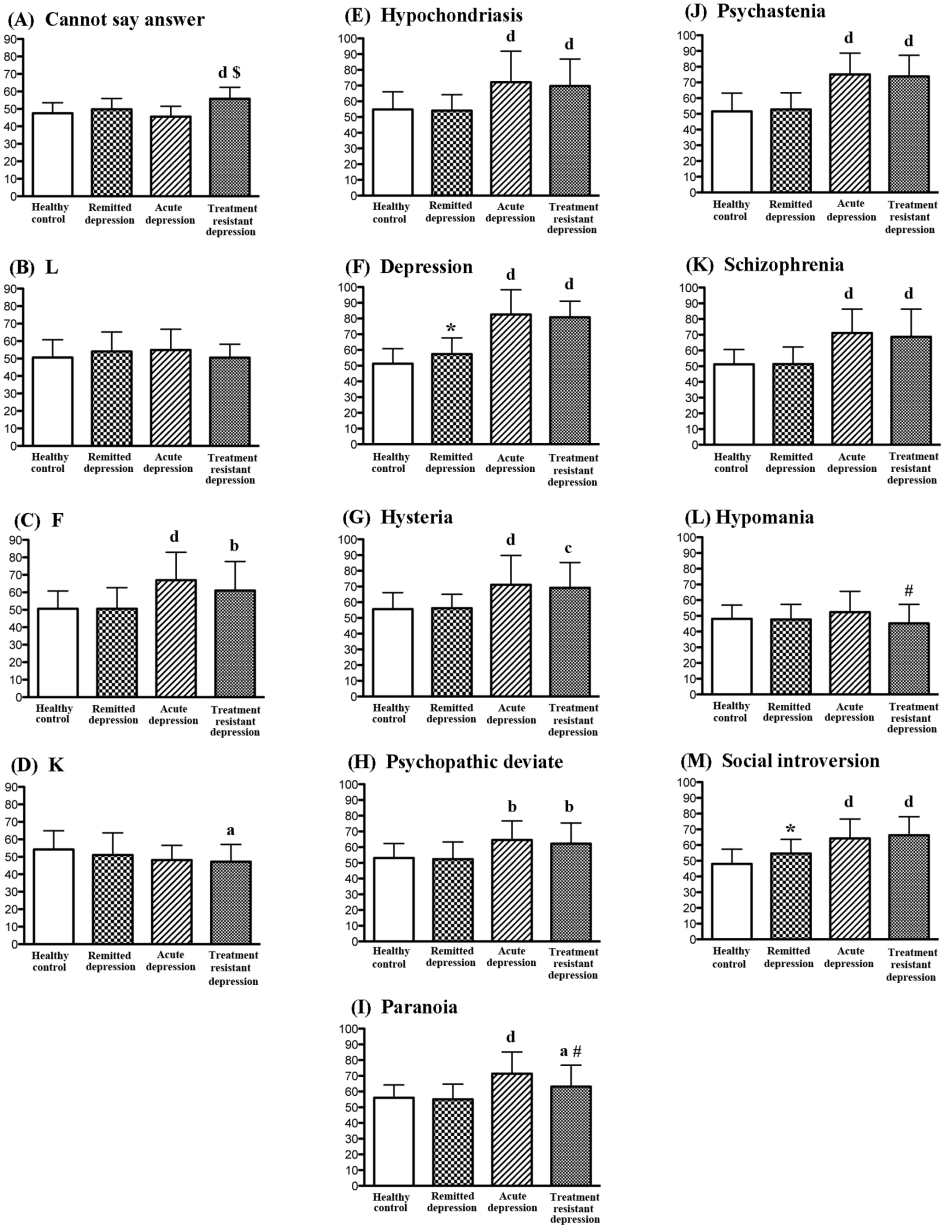
MMPI scales. Data are shown as mean ± SD. ^a^p<0.05, ^b^p<0.01, ^c^p<0.001, ^d^p<0.0001, as compared to the normal control group (ANOVA and post-hoc test). ^$^ p<0.0001 as compared to the acute depression group (ANOVA and post-hoc test). ^#^ P<0.05 as compared to the acute depression group (Student's t-test). * P<0.05 as compared to the normal control group (Student's t-test).

MANOVA for validity scales indicated a significant effect for group (F = 8.320, P<0.0001). Subsequent one-way ANOVA demonstrated statistical significance on the cannot say answer scale (F = 16.926, P<0.0001), F (F = 11.164, P<0.0001), K (F = 3.874, P = 0.0107), but not the L scale (F = 1.493, P = 0.2193). Post hoc testing (Scheffe's test) demonstrated that patients with treatment-resistant depression and those with acute depression showed significantly altered scores on the MMPI, relative to patients with remitted depression and healthy controls (**Figure 1**). Patients with treatment-resistant depression showed significantly higher scores in the cannot say answer scale compared with normal controls and patients with acute depression (P<0.0001).

### MMPI for clinical features

MANOVA for clinical scales indicated a significant effect for group (F = 7.236, P<0.0001). One-way ANOVA demonstrated statistical significances for hypochondriasis (F = 14.570, P<0.0001), depression (F = 77.949, P<0.0001), hysteria (F = 12.875, P<0.0001), psychopathic deviate (F = 9.668, P<0.0001), paranoia (F = 13.027, P<0.0001), psychasthenia (F = 37.567, P<0.0001), schizophrenia (F = 22.698, P<0.0001), and social introversion (F = 27.778, P<0.0001), but not hypomania (F = 2.078, P = 0.1059).

Patients with treatment-resistant depression and acute depression showed significantly altered scores on the MMPI relative to patients with remitted depression and healthy controls (**Figure 1**). However, we found a significant difference only in the cannot say answer scale between treatment-resistant depression and acute depression patients. We found no significant differences in any scales between remitted depression patients and healthy controls.

### Comparisons between the two groups

Since ANOVA and post hoc testing showed a trend for significance between patients with treatment-resistant depression and those with acute depression in some scales (paranoia, P = 0.0670; mania, P = 0.1074), we used Student's t-test to detect small differences between the two groups. We found statistically significant differences between treatment-resistant depression and acute depression on the paranoia (t = 2.141, P = 0.0369), and mania scales (t = 2.088, P = 0.0416) (**Figure 1**).

Similarly, since ANOVA and post hoc testing showed a trend for significance between patients with remitted depression and normal controls in some scales (depression, P = 0.1397; social introversion, P = 0.0744), we used Student's t-test to detect small differences between the two groups. We found a significant difference between remitted depression and normal controls on the depression (t = 2.577, P = 0.0117), and social introversion scales (t = 2.837, P = 0.0057) (**Figure 1**).

### Pessimism and optimism by MMPI

**Figure 2:**
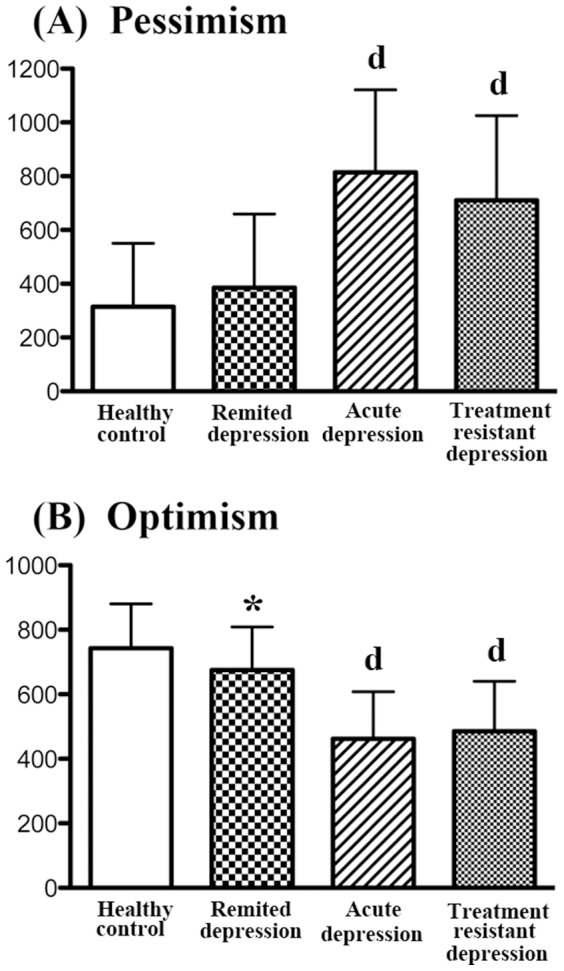
Pessimism and Optimism on the MMPI. Data are shown as mean ± SD. ^d^ p<0.0001 as compared to the normal control group (ANOVA and post-hoc test). * P<0.05 as compared to the normal control group (Student's t-test).

MANOVA for pessimism and optimism scales demonstrated a significant effect for group (F = 17.472, P<0.0001). One-way ANOVA highlighted statistically significant differences on pessimism (F = 26.958, P<0.0001) and optimism (F = 35.357, P<0.0001). Patients with treatment-resistant depression and acute depression showed significantly altered scores on the MMPI, compared with patients suffering remitted depression, or healthy controls (**Figure 2**).

Although post hoc testing failed to show significant changes between patients with remitted depression and normal controls on the optimism scale (P = 0.2572), we used Student's t-test to detect small differences between the two groups, and found a significant difference between remitted depression and normal controls in this trait (t = 2.116, P = 0.0372) (**Figure 2**).

## Discussion

This study found that patients with treatment-resistant depression or remitted depression exhibited psychological features in their MMPI profile, different to those in acutely depressed patients or normal controls. These data are supported by a previous study which showed that unipolar depression patients had a higher elevation in the depression scale, predominant over disturbances in the hypocondriasis, depression, hysteria, psychopathic deviate, paranoia, paychasthenia, schizophrenia and social introversion scales on the MMPI [8]. Another study showed that disturbances in unipolar depression, psychopathic deviation, paranoia, psychasthenia and schizophrenia predominanted over disturbances in hypochondriasis, hysteria and social introversion on the MMPI [17]. A more recent study recorded mean scores for hypochondriasis, depression, hysteria, psychotic deviate, paranoia, psychasthenia and schizophrenia as greater than 65 T in major depression using MMPI-2 [18].

A review by Gross et al., showed that many studies used the depression scale with cut-off scores over 65 or 70 T in MMPI, suggesting that the depression scale in the MMPI is moderately accurate for predicting depression [6]. Clinically, 65 T is 1.5 standard deviations over the mean. The percentage of depression scores above 65 or 70 T is 95 and 81 percent in acutely depressed patients, 94 and 88 percent in patients with treatment-resistant disease, 32 and 4 percent in remitted depression, and 8 and 5 percent in normal controls, respectively. Between the two groups, we found a significant difference between patients with remitted depression and normal controls in the depression scale over 65 T (Chi-square 8.432, p = 0.0037), but not over 70 T. Furthermore, depressed patients in remission showed a significant elevation in the depression scale when evaluated by Student's t-test, but not by ANOVA or post-hoc testing (Figure 1). Therefore, it is likely that small depressive traits are present but masked in remitted depression patients, even though they scored less than 7 on HAM-D. This is supported by a recent study which found depressed outpatients who were deemed to be in remission according to HAM-D [19].

Our study demonstrated that when using the MMPI, criteria other than the depression scale were useful to understand the psychological status of depressed patients. The MMPI profile of depression is characterized by elevations on the depression, psychasthenia and schizophrenia scales [5], [17]. The psychasthenia scale assesses ruminative worry and is called the anxious personality scale, indicating generalized feelings of anxiety and discomfort. The schizophrenia scale assesses cognitive confusion or bizarreness. A previous study using two-high scales proposed seven MMPI personality profiles in depressed inpatients, namely, depression plus schizophrenia, depression plus psychasthenia, depression plus psychopathic deviate, depression plus hysteria, schizophrenia plus psychopathic deviate, schizophrenia plus psychasthenia and schizophrenia plus hypomania [11], indicating that depression and schizophrenia scales predominante psychopathic deviate or psychasthenia scales, and to a lesser extent, hysteria and hypomania scales, on the MMPI. Another study reported that psychopathic deviate plus schizophrenia scales were frequently found in depressed patients with personality disorders [10]. However, in our results, the depression scale took precedence over schizophrenia or psychasthenia, and to a lesser extent, hypochondriasis and hysteria in patients with acute and treatment-resistant depression (Figure 1).

Influence of neurotic based mechanisms was indicated by high scores in all three neurosis related MMPI profiles (hypochondriasis, depression and hysteria). Hypochondriasis assesses somatic complaints, while hysteria assesses a state of repression, low coping skills and excessive response to strong trauma. Hysteria correlates with a high body resistance. Patients with somatization disorder showed high hypochondriasis and hysteria compared with primary depression [20]. Another study reported differences between dysthymia and major depression for scales in hypochondriasis and hysteria, and to a lesser extent, depression, in the MMPI profile [9]. The theorized presence of neurotic mechanisms in patients with treatment-resistant depression seems to be justified, since these patients often feel frustrated with the difficulty of adhering to regular work or social schedules. This study demonstrated that the percentage of the neurotic triad evaluating all three items over 65 or 70 T was 71 and 47 percent in acutely depressed patients and 44 and 38 percent in patients with treatment-resistant depression, respectively. We found a statistically significant difference between patients with treatment-resistant depression and those with acute depression over 65 T (Chi-square 3.905, p = 0.0481), but not over 70 T. In contrast to our expectation, patients with treatment-resistant depression suffered equally or lower levels of neurotic pathophysiology, compared with those suffering acute depression.

A previous study demonstrated that psychopathic deviate plus paranoia, or psychopathic deviate plus hypomania at a T-score above 70 in the MMPI, met the criteria for personality dysfunction [21]. The psychopathic deviate scale indicates anti-social behavior, hostility or conflict. Here, the percentage of patients with a psychopathic deviate plus paranoia pattern over 65 or 70 T, was 42 and 33 percent in acutely depressed patients and 29 and 30 percent in patients with treatment-resistant depression respectively. The percentage of a psychopathic deviate plus hypomania pattern over 65 or 70 T was 6 percent in acutely depressed patients and 10 percent in patients with treatment-resistant depression, in both groups. These were no statistical differences between any of the two examined groups by Chi-square method.

The patients with treatment-resistant depression showed weak alterations on paranoia and hypomania scales compared to those with acute depression by Student's t-test (Figure 1). Since the paranoia scale assesses sensitivity to personal communication, low paranoia levels, although still above normal controls, indicate impaired sensitivity in personal communication. Low hypomania scores point to low activity. It is likely that patients with treatment-resistant depression exhibit low social activity, compared with acutely depressed patients.

For validity scales scores, ANOVA and post-hoc testing demonstrated that the cannot say answer scale was significantly increased in treatment-resistant depression patients, relative to acutely depressed patients (Figure 1). This seems to reflect enhanced difficulty in decisions making in this cohort, compared with other groups. Additionally, the K scale was significantly decreased in patients, but only in those with treatment-resistant depression (Figure 1). Since low K scores denote a tendency for self-criticism, patients with treatment-resistant depression appear to be introversive. However, F scales were significantly increased in both treatment-resistant and acute depression patients, compared with normal controls (Figure 1). The F scale is an atypical response and is thought to reflect an exaggeration of complaints, poor thinking ability or inattention. On this scale, patients with treatment-resistant depression are similar with those with acute depression.

Recently we reported on the psychological profiles of patients with treatment-resistant depression, namely, low scores for reward dependence and cooperativeness on the (Temperament and Character Inventory) TCI and low scores for openness on the NEO Personality Inventory, in contrast with depressed patients [22], [23]. It is conceivable that these alterations share some relationship with our current results.

Patients in remitted depression showed significantly higher rises in depression or social introversion scores on the MMPI, compared with normal controls when evaluated by Student's t test, and a trend for significance by ANOVA and post-hoc testing (Figure 1). High social introversion assesses introversive tendencies or withdrawal. This is a unique characteristic of patients in remitted depression.

Looking at pessimism and optimism, patients with treatment-resistant and acute depression demonstrated higher pessimism and lower optimism than normal controls (Figure 2). Although the explanatory styles for pessimism and optimism in the two groups are different from normal controls, the degrees of alteration were not significantly different between the two groups, indicating similar levels in patients with treatment-resistant and acute disease. Therefore, these characteristics are not unique to patients with treatment-resistant depression.

Patients with remitted depression showed lower optimism compared with normal controls when evaluated by Student's t-test (Figure 2). The exact cause of this marginally lower optimism is unknown, but seems noteworthy. Our recent study revealed that patients in remitted depression who had suffered a single depressive episode, had higher scores of harm avoidance on the TCI, compared with healthy controls [22]. This lower optimism on the MMPI and higher harm avoidance on the TCI may well be interrelated pathological mechanisms in these patients.

Self-directedness on the TCI increased during remission from major depression [24]. Previous studies showed that patients with remitted depression exhibited low self-directedness as well as high harm avoidance on the TCI [25]–[27]. However, it is conceivable that these traits are related to the recurrence of depressive episodes since the remitted depression patients in these studies were characterized by recurrent episodes. Other studies on remitted depression with patients suffering single episodes, failed to show any changes using the Maudsley Personality Inventory [28] or the NEO [23]. Studies therefore should distinguish whether their remitted depression patients suffered single or recurrent episodes of depression. Considering these differences, the characteristics identified in this study using remitted patients after a single episode of depression, are unique and useful for future studies.

This study has a number of limitations. First, the study is of a cross-sectional design, making it difficult to accurately determine the degree to which a participant's depressed state influences their responses. In addition the recruits were volunteers with potentially limited generalisability. Also, there was no adjustment for potential confounding factors, such as gender difference, duration of treatment, duration of depressive state and education. This means that the independence of associations cannot be assumed, and that negative associations need to be viewed with caution because of the relatively small sample size. Finally, the remission and control groups differed in that the former groups had suffered, and received treatment for depression. It is therefore possible that the remitted group could still be experiencing sub-case symptomatology, since their depression scores fell between 3 and 7 on the HAM-D (Table 1).

In conclusion, there are subtle but important alterations on the MMPI, which may underpin the hidden psychological features of patients with both remitted and treatment-resistant depression.
